# Structural and functional characteristics of cGMP-dependent methionine oxidation in *Arabidopsis thaliana* proteins

**DOI:** 10.1186/1478-811X-11-1

**Published:** 2013-01-05

**Authors:** Claudius Marondedze, Ilona Turek, Brian Parrott, Ludivine Thomas, Boris Jankovic, Kathryn S Lilley, Chris Gehring

**Affiliations:** 1 Division of Chemical and Life Sciences and Engineering, King Abdullah University of Science and Technology, Thuwal, 23955-6900, Saudi Arabia; 2 Computational Bioscience Research Center, King Abdullah University of Science and Technology, Thuwal, 23955-6900, Saudi Arabia; 3 Cambridge Centre for Proteomics, Cambridge Systems Biology Centre, Department of Biochemistry, University of Cambridge, Tennis Court Road, Cambridge, CB2 1QR, UK

**Keywords:** Methionine oxidation, Reactive oxygen species, 3,5-cyclic guanosine monophosphate, Tandem mass spectrometry-based proteomics, *Arabidopsis thaliana*

## Abstract

**Background:**

Increasing structural and biochemical evidence suggests that post-translational methionine oxidation of proteins is not just a result of cellular damage but may provide the cell with information on the cellular oxidative status. In addition, oxidation of methionine residues in key regulatory proteins, such as calmodulin, does influence cellular homeostasis. Previous findings also indicate that oxidation of methionine residues in signaling molecules may have a role in stress responses since these specific structural modifications can in turn change biological activities of proteins.

**Findings:**

Here we use tandem mass spectrometry-based proteomics to show that treatment of *Arabidopsis thaliana* cells with a non-oxidative signaling molecule, the cell-permeant second messenger analogue, 8-bromo-3,5-cyclic guanosine monophosphate (8-Br-cGMP), results in a time-dependent increase in the content of oxidised methionine residues. Interestingly, the group of proteins affected by cGMP-dependent methionine oxidation is functionally enriched for stress response proteins. Furthermore, we also noted distinct signatures in the frequency of amino acids flanking oxidised and un-oxidised methionine residues on both the C- and N-terminus.

**Conclusions:**

Given both a structural and functional bias in methionine oxidation events in response to a signaling molecule, we propose that these are indicative of a specific role of such post-translational modifications in the direct or indirect regulation of cellular responses. The mechanisms that determine the specificity of the modifications remain to be elucidated.

## Findings

**Figure 1:**
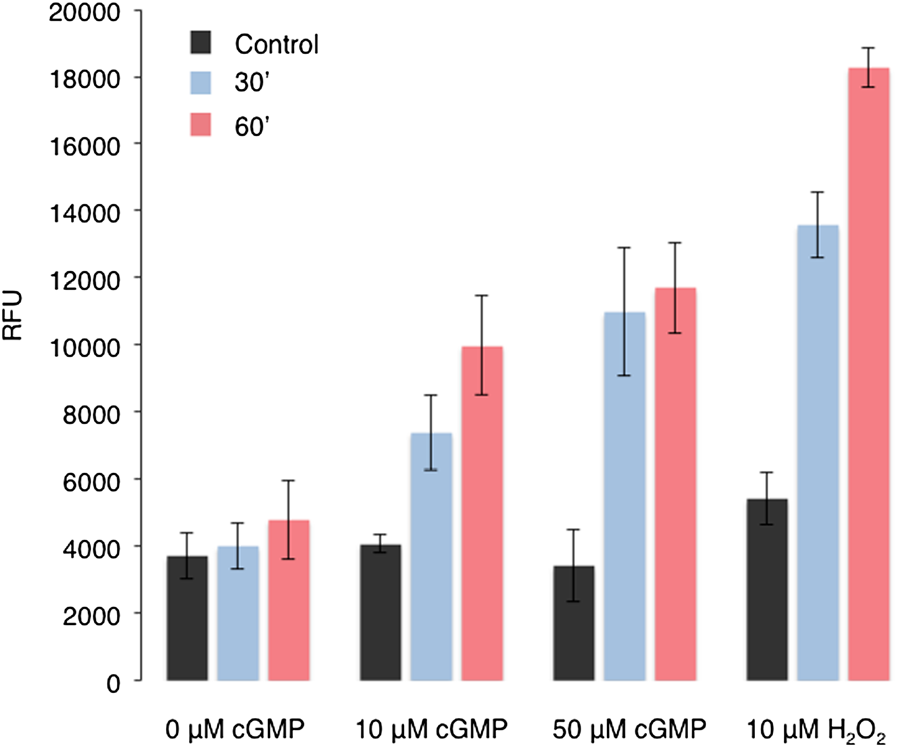
**Protein oxidation assay.** OxiSelect™ Intracellular ROS assay kit (Cell Biolabs, Inc. San Diego, CA) was used in the *in vivo* oxidation experiments according to the assay protocol provided by the manufacturer. Cultured Arabidopsis (Col-0) cells were placed in a black bottom 96-well cell culture plate for 2 h in a shaking incubator. The 2^′^,7^′^-dichlorofluorescein diacetate/media solution was added to the cells prior to incubation at 37°C for 1 h. The dye-loaded cells were then treated with 10 μM or 50 μM of cGMP or H_2_O_2_. Fluorescence in the cells was measured at 30 and 60 min post-treatment at 480/530 nm using a PHERAstar *FS* microplate reader (BMG Labtech GmbH, Germany) and the values plotted. Each bar represents data from 3 biological replicates (n = 3), the bars are the standard errors. Treatment with 8-Br-cGMP at the final concentration of 50 μM induces statistically significant differences of the means at p = 0.05 using a two-sample *t*-test.

**Figure 2:**
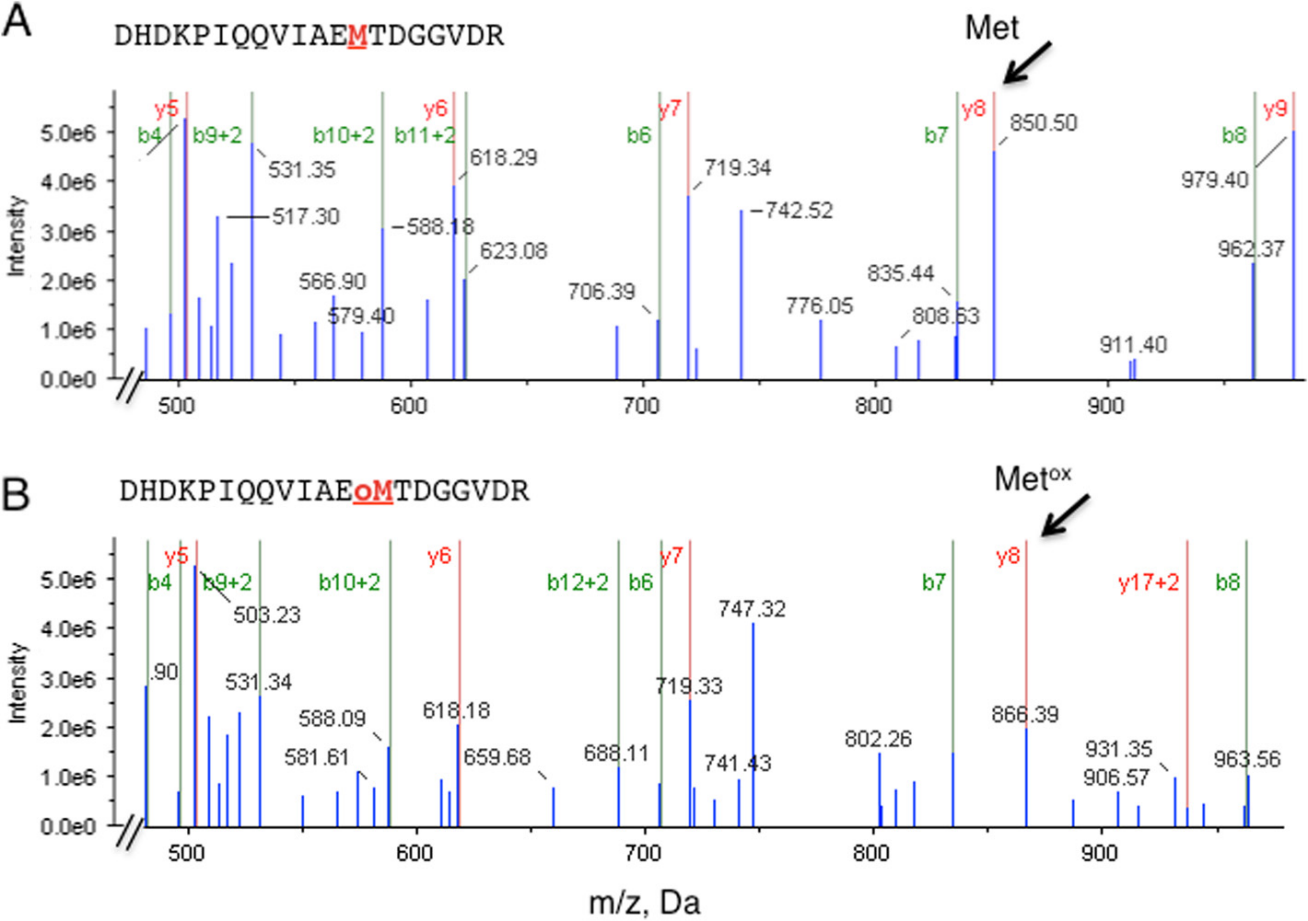
**MS/MS spectra of ADH1 containing non-oxidised (A) and oxidised (B) methionine residues.** Three biological replicates of 10 μM 8-Br-cGMP-treated cells and H_2_O mock treated controls were collected at 0, 30 and 60 min. Proteins were precipitated using 10% (w/v) trichloroacetic acid in acetone, re-solubilised in 7 M urea, 2 M thiourea and 4% (w/v) CHAPS, reduced, alkylated and trypsin digested. Peptides were fractionated by cation exchange chromatography. Methionine oxidised peptides were enriched using TiO_2_ beads and re-suspended in 5% (v/v) acetonitrile and 0.1% (v/v) formic acid prior to identification and quantitation by LTQ Orbitrap coupled with a nanoelectrospray ion source. Peptides (5 μL) were injected onto a 50 mm × 0.3 mm Magic C18AQ column. The top 10 precursor ions were selected with a resolution of 60,000 for fragmentation using normalized collision-induced dissociation set at 35.0. Spectra were searched against TAIR using MASCOT, with a precursor mass tolerance of 10 ppm, a fragment ion mass tolerance of 0.3 Da, one missed cleavage, carbamidomethyl cysteine residues as fixed modification and oxidation and dioxidation of methionine residues as variable modifications. Proteins with a score > 95% were considered positively identified (corresponding score ≤ 31). Spectra were further processed with the Scaffold*™* software using the “Trans-Proteomic Pipeline” algorithm (threshold 95%). Oxidised Met residues showed an increase in mass/charge ratio (*m/z*) of 15.9994. Arrows show Met residues at position 13 in the fragment DHDKPIQQVIAEMTDGGVDR of AT1G77120 before oxidation (*m/z* ratio 850.3723) (**A**) and after oxidation (*m/z* ratio 866.3673) (**B**).

The debate of whether methionine (Met) oxidation of proteins is a purely chemical consequence of cellular oxidative damage or a protective mechanism against oxidative damage, or indeed a post-translational modification that can act as a specific cellular signal and/or response, is ongoing [1-3]. To shed light on this question we treated Arabidopsis suspension culture cells with the cell permeant second messenger analogue 8-bromo 3,5-cyclic guanosine monophosphate (8-Br-cGMP). Cyclic GMP has a signaling role in many plant responses, including responses to light [4], hormones [5-8], signaling peptides [9], salt and drought stress [10,11], ozone, and defence responses [12-14]. Given that cGMP is not an oxidising agent and does not induce protein Met oxidation *in vitro* (Additional file 1), we tested if cGMP causes protein oxidation *in vivo*. To this end we used an OxiSelect™ Intracellular ROS Assay Kit (Cell Biolabs, Inc.) and show that cGMP can cause protein oxidation (Figure 1). We therefore conclude that any protein Met oxidation event resulting from cGMP treatment is most likely the result of direct or indirect cellular processes. To further characterise cGMP-dependent Met oxidation *in vivo*, a proteomic analysis was performed on *A. thaliana* (ecotype Col-0) cell suspension culture grown in Murashige and Skoog medium [15] following treatment with 8-Br-cGMP at the final concentration of 10 μM. Three biological replicate samples were collected at 0, 30 and 60 minutes post-treatment. Total soluble proteins were extracted [16] and processed for tandem mass spectrometric identification of peptides containing oxidised Met residues (for methods see legend to Figure 2). Since our experimental protocol included a TiO_2_ enrichment step, usually applied for enrichment of phosphopeptides, we also tested to what extent peptides containing oxidized Met residues are enriched in samples subjected to the TiO_2_-based enrichment step either in the presence of absence of DHB (2,5-dihydroxybenzoic acid). In both cases the enrichment led to significant increase in ratio of spectra assigned to oxidised Met peptides to all assigned spectra (Table 1). Moreover, the presence of DHB in the TiO_2_-based enrichment step enhanced further increase in the number of oxidized peptides identified as compared to oxidized Met peptides enriched in the absence of DHB. This is consistent with a report that shows that Met oxidised peptides co-enrich with phosphopeptides because the affinity for the TiO_2_ (in the presence of DHB) is stronger in oxidised as compared to non-oxidised isoforms [17].

**Table 1:** ^**†**^**Enrichment of methionine oxidized peptides (oxMet) using TiO**_**2**_**with and without DHB**

	**Without enrichment**				**TiO**_**2**_**enrichment without DHB**				**TiO**_**2**_**enrichment with DHB**			
	**R1**	**R2**	**R3**	**R4**	**R1**	**R2**	**R3**	**R4**	**R1**	**R2**	**R3**	**R4**
Assigned spectra	123109	67688	42897	89362	38403	56545	14450	4288	28092	11880	8567	3812
Assigned spectra oxMet pep.	3073	288	49	3930	15803	4273	6303	424	26913	4370	6586	1318
% oxMet	2.5	0.4	0.1	4.4	41.2	7.6	43.6	9.9	95.8	36.8	76.9	34.6

^†^The cell suspension culture was treated with 10 μM H_2_O_2_ for 30 minutes. The experimental procedures are as described in the legend to Figure 2.

In our proteomic analysis we considered a peptide as containing oxidised Met residue when it was identified with high confidence (≥ 95%) in at least two biological replicates. A total of 385 cGMP-dependent methionine oxidised proteins were identified (Additional file 2, tab “AF1”). Assigned spectral counts (Additional file 2, tab “AF2”) were used to estimate the relative ratio of peptides containing oxidized Met residue(s) as compared to total number of peptides identified in the sample.

An example of a tandem mass spectrometry result demonstrating oxidative modification of TiO_2_-enriched peptides extracted from 8-Br-cGMP-treated cells is shown in Figure 2. Peptides containing single oxidised Met residue show an increase in mass to charge (*m/z*) ratio of 15.9994 that corresponds to the average mass of an oxygen atom. For example, the peptide fragment (DHDKPIQQVIAEMTDGGVDR) of the alcohol dehydrogenase 1 (AT1G77120) in non-oxidised form has the *m/z* ratio of 850.3723 (Figure 2A), while after oxidation of Met residue, the *m/z* ratio shifts to 866.3673 (Figure 2B).

**Figure 3:**
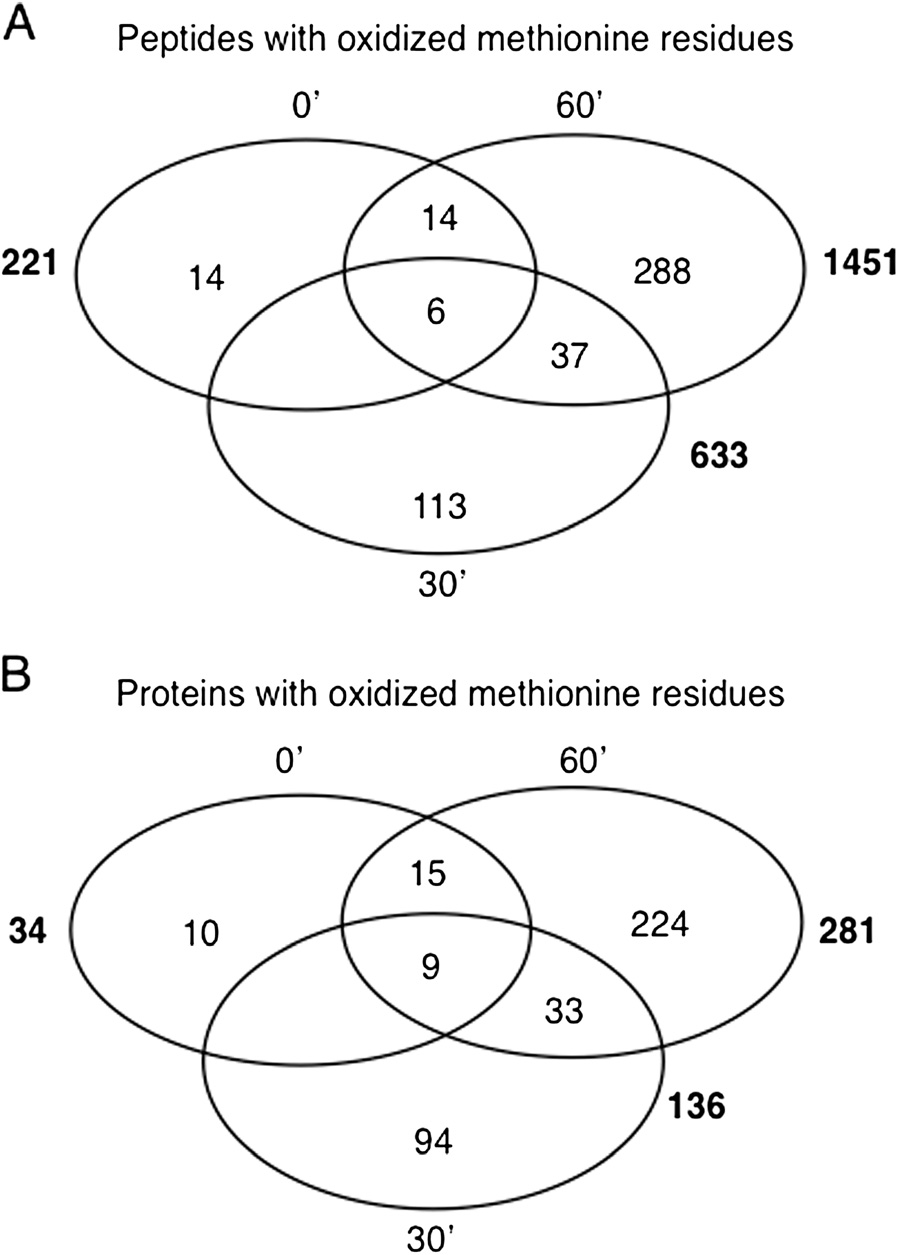
**Quantitative representation of peptides (A) and proteins (B) containing oxidised Met residues identified by mass spectrometry.** The total numbers of peptides and proteins containing at least one oxidised Met residue identified by LC-MS/MS in protein samples extracted from *A. thaliana* cells treated with 10 μM 8-Br-cGMP and collected at 0, 30 and 60 min post-treatment were analysed. Proteins matching the peptides were identified by searching against TAIR10 database using MASCOT and TPP algorithm, and only proteins within a 95% confidence threshold were considered. The total number of peptides (**A**) or proteins (**B**) are indicated outside the Venn diagram (in bold), the numbers inside are the unique peptides or proteins identified at each time point. Fourteen, 113 and 288 peptide fragments containing at least one oxidised Met appear only at either 0, 30 or 60 min. This corresponds to an increase in the total number of time point specific Met oxidised proteins from 10 to 94 and then to 224 (**B**).

Further, we identified peptides with oxidised Met that occurred in all three biological replicates at different time points. We noted an increase in the total number of peptides containing residues of oxidised Met after cGMP treatment from 221 to 633 and then 1451 at 0, 30 and 60 minutes, respectively (Figure 3A and Additional file 2, tab “AF2”). These numbers represent 1.4%, 19.4% and 13%, respectively, of the total number of peptides identified at each time point. Thus, the percentage of Met oxidised peptides identified is the highest at 30 minutes. In addition, the numbers of oxidised Met peptides detected at each time-point suggest that the total number of oxidised Met residues increased nearly 3-fold during the first 30 minutes of treatment and 7-fold after 60 minutes of treatment (Additional file 2, tab “AF2”). Of these redundant peptide fragments containing oxidized Met, 14 at 0 minutes, 113 at 30 minutes and 288 at 60 minutes were unique for each time-point. The total Met oxidised peptides correspond to 34 (at 0’), 136 (at 30’) and 281 (at 60’) Arabidopsis proteins, from which 10, 94, and 224 identified oxidized Met proteins are unique for each time-point, respectively (Figure 3B). This finding implies either that cGMP-dependent Met modifications are reversible and/or that some of the modified proteins have undergone proteolysis.

**Figure 4:**
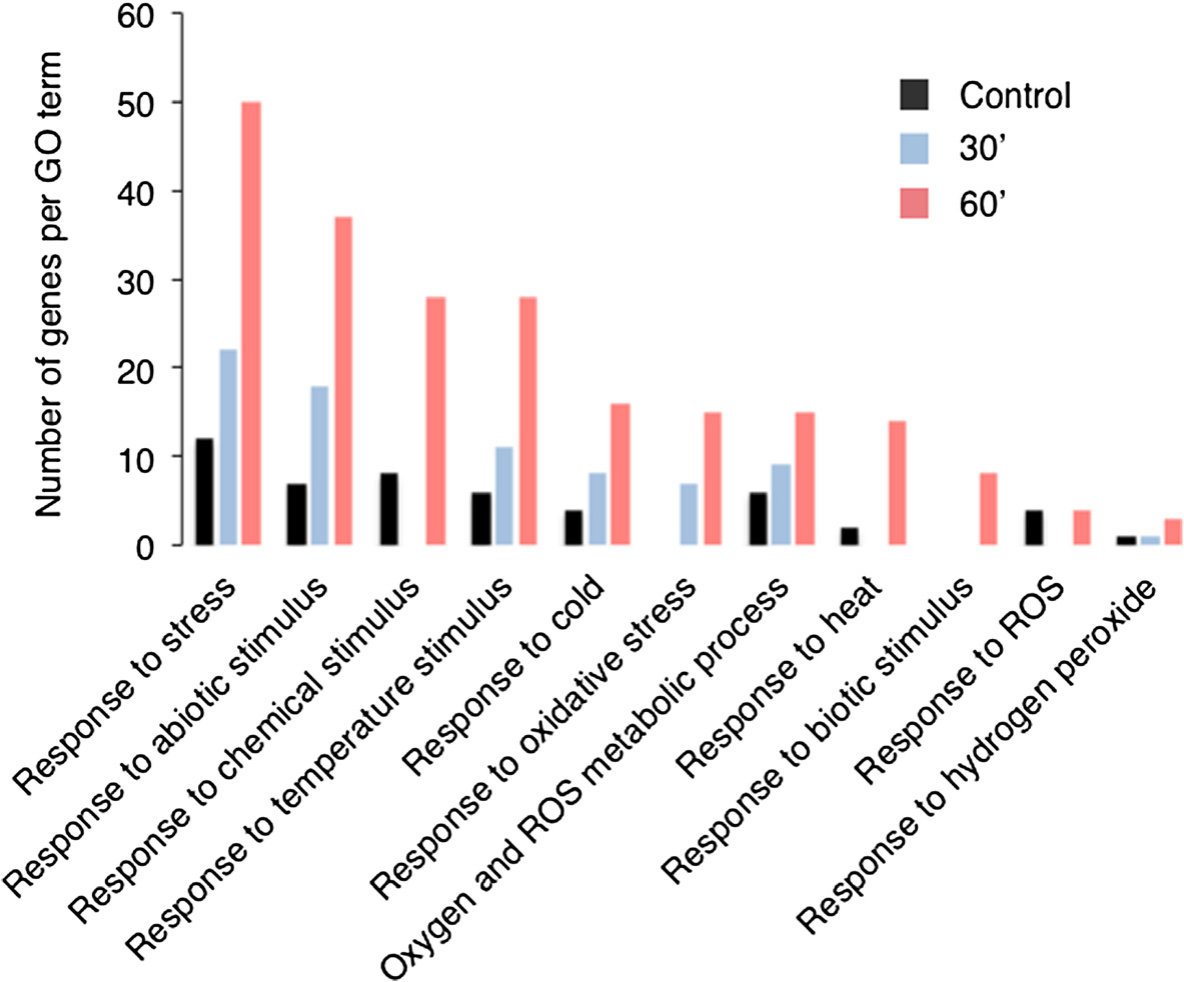
**Enriched gene ontology (GO) terms (adjusted p-value < 1.00e**^**-02**^**).** Unique proteins containing oxidised Met identified in cGMP-treated samples were used to search for GO term enrichment using Fatigo^+^ ( http://babelomics3.bioinfo.cipf.es/) and selected significantly enriched terms are represented. A significant increase in the enrichment of GO terms over time was observed as well as an increase in the number of genes in these GO categories.

Gene ontology (GO) analysis (Fatigo^+^; http://babelomics3.bioinfo.cipf.es/) [18] of the unique proteins containing oxidised Met residues was undertaken. The result shows a significant enrichment of GO terms (adjusted p-value < 1.00e^-02^) in categories including 'response to stress', 'response to abiotic stimuli', 'response to oxidative stress', and 'response to oxygen and ROS metabolic process' (see Additional file 2, tab “AF3”). Moreover, the gene number in these GO categories also increased over time (Figure 4). It therefore appears that the application of a membrane-permeable analogue of the second messenger cGMP induces Met oxidation in a set of proteins that are over-represented in specific functional groups.

Proteins enriched in the GO categories: 'response to oxidative stress', 'response to ROS', 'response to oxygen', and 'ROS metabolic process' showed four main patterns. (1) Loss of peptides or proteins containing oxidised Met residue(s) and reduction in the number of peptide copies after treatment, e.g. glyceraldehyde-3-phosphate dehydrogenase (AT1G13340; AT3G04120) and 60S acidic ribo-somal protein (AT2G27720). Loss of Met oxidised peptides can occur due to degradation of copies of proteins with oxidised Met residues or the reduction of oxidised Met residues. (2) Increase in the number of oxidised peptide fragments after treatment, e.g. peroxidase (AT2G22420; AT4G08770). This may imply that cGMP either indirectly induces over-expression of specific proteins with peptide fragments susceptible to oxidation or preferentially induces oxidation of Met residues in specific proteins without necessarily inducing transcription and/or translation. (3) New peptides detected with Met residues oxidised after treatment, e.g. ATP synthase (AT5G08670), and (4) multiple Met residues becoming oxidised after treatment, e.g. in the heat shock protein 70 (AT3G12580) (Additional File 2, tab “AF1”).

A total of 299 proteins (78% of all unique proteins containing oxidised Met residues) comprised only one oxidised Met residue, while 86 proteins (22% of all unique proteins containing oxidised Met residues) had ≥ 2 oxidised Met residues (Additional file 2, tab “AF4”). Of those multi-oxidised 86 proteins 10 proteins had ≥ 4 Met residues oxidised (Additional file 2, tab “AF5”). The ‘low expression of osmotically responsive gene 1’ (AT1G56070), ‘tubulin 9’ (AT4G20890), ‘heat shock protein 70’ (AT5G02500) and ‘male gametophyte defective 1’ (AT2G21870) contain the largest number of oxidised residues, representing 16%, 23%, 29% and 50%, respectively, of their total Met content (Table 2 and Additional file 2, tab “AF5”). It is of interest that heat shock proteins AT5G02500, AT3G12580 and AT5G09590 each had at least four oxidised Met residues.

**Table 2:** cGMP-dependent proteins with multiple methionine residues oxidised and their enriched GO terms

**Accession No.**	**Description**	*OxMet/total Met residues	**Annotations**	**Enriched GO term(s)**
AT1G56070	Low expression of osmotically responsive gene 1	5/38	RC, translational elongation	RTS
AT2G21870	Male gametophyte defective 1	5/10	Co^2+^, Cu^2+^ and Zn^2+^ binding	
AT4G20890	Tubulin 9	5/22	GTP binding, GTP catabolic process, RCa	
AT5G02500	Heat shock protein 70	5/17	Responses to heat, bacterium, H_2_O_2_ and HLI, RCa, RTS, RV	RTS, RV
AT3G12580	Heat shock protein 70	4/16	Responses to heat, bacteria, H_2_O_2_, HLI, RCa, RTS, RV	RTS, RV
AT3G27240	Cytochrome C1 family	4/8	Heme binding, Fe^2+^ binding	
AT4G02930	GTP binding elongation factor Tu family protein	4/14	ATP, Co^2+^ and Zn^2+^ binding, RCa	
AT1G24310	Unknown protein	4/12		
AT1G27390	Translocase outer membrane 20	4/8	Metal ion binding	
AT4G10480	Nascent polypeptide-associated complex	4/10	Transcription regulation and mitochondrial translocation	

HLI- high light intensity, RC- response to cold, RCa- response to cadmium ion, RTS- response to temperature stimulus (GO:0009266), RV- response to virus (GO:0009615), adjusted p < 1.78e^-2^. ^*^Ox- oxidised.

The protein with the highest number of Met oxidised peptide fragments in response to cGMP is the methylesterase PCR A (AT1G11580) with 235 oxidised fragments representing ≈ 30% of the identified peptides (Table 3 and Additional file 2, tab “AF5”). Among the proteins with peptides show the greatest extend of Met oxidation, peroxidase superfamily protein (AT2G22420) showed the highest ratio of Met oxidised peptides to all identified peptide fragments of the protein (114 out of 160), representing ≈ 70% of total peptides detected and assigned to this protein. Localised in the cytosol, this family of proteins is mainly involved in oxidation-reduction processes and responses to oxidative stress. In addition to ROS being generated by activation of a plasma membrane NADPH oxidase [19], an extracellular cell wall peroxidase is also involved in the biosynthesis of H_2_O_2_[20] and plays an important role in plant resistance to pathogens [21]. A previous study using antisense expression of a French bean peroxidase cDNA in Arabidopsis showed a reduction in mRNA level of peroxidase (AT3G49120), which in turn led to a reduction in the oxidative burst and eventually a reduced resistance to fungal and bacterial pathogens [22]. The authors hypothesized that peroxidases have a role in sustaining and/or initiating ROS that signal early defence responses in plants. Given that peroxidases themselves are highly susceptible to Met oxidation, and that this modification limits their activity, it suggests a negative feedback mechanism and links Met oxidation to the control of cellular redox balance. It has also been reported that O_3_ and NO, both oxidising agents, induce transcriptional activation of scavenger-encoding genes, like *alternative oxidase* and *glutathione peroxidase*[12]. Furthermore, oxidation of Met residues has been shown to target both specific functional domains and consequently modify functional characteristics, e.g. in cytochrome c [23] and peroxidases [24], as well as regions outside functional domains, like in the nascent polypeptide-associated complex where modifications do not seem to affect functionality.

**Table 3:** Highly methionine oxidised proteins after cGMP treatment

**Accession No.**	**Description**	**% of oxidised fragments**	**Annotations**	**Enriched GO term(s)**
AT2G22420	Peroxidase superfamily protein	71.3	Oxidation-reduction process, response to oxidative stress	
AT5G12250	β-6-tubulin	62.6	Response to salt stress, RC	
AT4G10480	NAC	50.9	Transcription regulation and mitochondrial translocation	
AT3G15950	NAI2	41.8	Response to salt stress	
AT2G05710	Aconitase 3	35.0	Response to ABA stimulus, oxidative and salt stress, RCa	
AT5G02500	Heat shock protein 70	33.6	Defense response to bacteria and fungus, response to heat, and virus, RCa, RC	RAS, RS
AT2G21660	Cold, circadian rhythm and RNA binding 2	32.7	Regulation of stomatal movement, response to osmotic and salt stress, RCa, RC	RAS, RS
AT1G11580	Methylesterase PCR A	30.8	Metabolic process, negative regulation of catalytic activity	
AT1G77120	Alcohol dehydrogenase	18.7	Cellular respiration, oxidation-reduction process, response to hypoxia, osmotic stress and salt stress, RCa	RAS, RS
AT1G53240	Mitochondrial malate dehydrogenase	16.9	Oxidation-reduction process, defense response to bacteria, response to salt stress, RCa, RC	

*NAC* Nascent polypeptide-associated complex, NAI2- similar to TSK-associating protein 1, *RC* Response to cold, *RCa* Response to cadmium ion, *RAS* Response to abiotic stimulus (GO:0009628), *RS* Response to stress (GO:0006950), adjusted p < 4.15e^-2^.

Given that Met oxidation can profoundly alter cellular responses, the question is if there is evidence for site selectivity and if so, what determines it. In order to address this question, we have subjected all 385 proteins with one or several oxidised Met to further analysis and noted the following. Firstly, the average Met frequency in the set of proteins containing oxidised Met is 0.027 as compared to the other amino acids (AA) in the complete proteome where it is 0.025 [25], and hence is nearly the same; we also noted that none of the 50 Arabidopsis proteins with the highest frequency of Met occurrence (0.09) contained any oxidised Met residues. This indicates that Met frequencies in proteins *per se* are not a factor that determines increased oxidation. Secondly, of the 385 proteins containing at least one modified Met residue, only eight residues were on the N-terminus, and of the 150 double Met (-MM-) residues, five proteins had both residues oxidised and eight proteins - only one. Thirdly, of the 575 oxidised Met residues detected, 75 had a glutamic acid (Glu) and 68 had an aspartic acid (Asp) as an immediate C-terminal neighbour and this bias is likely, at least in part, due to preferential enrichment of these AAs by TiO2 [26], however we also find the uncharged alanine (Ala: 47) enriched on the C-terminus. The most frequent N-terminal neighbours are glutamic acid (Glu: 67) and again the uncharged alanine (Ala: 65). The least frequent C- and N-terminal neighbours are tryptophan (Trp: 2; 0, respectively) and cysteine (Cys: 1; 2, respectively). We further compared the observed frequency of amino acids flanking Met positions to their theoretically expected values implied from the analysis of overall AA frequencies in the entire Arabidopsis proteome [25]. This process involved counting all of the occurrences of AAs in the proteome and establishing their relative frequency in the proteome, and the analysis was done in Matlab (Version R2010b). We note that, while under-represented (i.e. the observed frequency in positions flanking Met is lower than their average in the entire proteome), leucine and serine are the most frequent flanking AAs. In turn, Met flanking a Met is 35% over-represented on the N-terminal side and 25% over-represented on the C-terminal side. In contrast, oxidised Mets have much reduced relative preference for Met (-14% on N-terminus and -44% on C-terminus), albeit based on a very limited sample. Other under-represented flanking AAs are cysteine (Cys) and proline (Pro).

The rapidly evolving field of redox proteomics provides new evidence supporting the notion that oxidation of Met residues may have a great impact on protein activity, regulation of biochemical pathways and cellular function in response to changing environmental conditions. This is consistent with the observed Met oxidation accumulation in plants under low temperature conditions and the fact that plant methionine sulfoxide reductase (MSR) confers increased tolerance to freezing [27]. It is also conceivable that the differential oxidation footprint of Met is a result of different susceptibility depending e.g. on the conformation of the protein or on differential access for repair of the MSR to different proteins or protein domains. Furthermore, our results are an indication that many of the Arabidopsis proteins involved in modulating the level of reactive oxygen species (ROS), including ROS-scavenging and ROS-producing proteins [19], may - at least in part - be regulated by oxidation of their Met residues.

## Competing interests

The authors declare that they have no competing interests.

## Authors’ contributions

CG conceived the project. CM, IT, BP, LT and KSL designed and performed the experiments, BJ performed the bio-informatics analyses, and CG wrote the manuscript with critical input from all authors. All authors read and approved the final manuscript.

## Supplementary Material

Additional file 1 **The file contains the list of methionine oxidised peptides and their assigned spectral counts following *in vitro *cGMP treatment experiment.** Click here for file

Additional file 2 The file contains the lists of all methionine oxidised proteins and fragments and the result of the gene ontology analysis. Click here for file

## References

[B1] Hoshi T Heinemann S Regulation of cell function by methionine oxidation and reduction J Physiol 2001 531 1 11 10.1111/j.1469-7793.2001.0001j.x 11179387 PMC2278441

[B2] Stadtman ER Moskovitz J Levine RL Oxidation of methionine residues of proteins: biological consequences Antiox Redox Sign 2003 5 577 582 10.1089/152308603770310239 14580313

[B3] Hardin SC Larue CT Oh MH Jain V Huber SC Coupling oxidative signals to protein phosphorylation via methionine oxidation in Arabidopsis Biochem J 2009 422 305 312 10.1042/BJ20090764 19527223 PMC2782308

[B4] Neuhaus G Bowler C Hiratsuka K Yamagata H Chua NH Phytochrome-regulated repression of gene expression requires calcium and cGMP EMBO J 1997 16 2554 2564 10.1093/emboj/16.10.2554 9184203 PMC1169867

[B5] Pharmawati M Billington T Gehring CA Stomatal guard cell responses to kinetin and natriuretic peptides are cGMP-dependent Cell Mol Life Sci 1998 54 272 276 10.1007/s000180050149 9575339 PMC11147171

[B6] Kwezi L Meier S Mungur L Ruzvidzo O Irving H Gehring C The Arabidopsis thaliana brassinosteroid receptor (AtBRI1) contains a domain that functions as a guanylyl cyclase in vitro PLoS One 2007 2 e449 10.1371/journal.pone.0000449 17520012 PMC1867859

[B7] Kwezi L Ruzvidzo O Wheeler JI Govender K Iacuone S Thompson PE Gehring C Irving HR The phytosulfokine (PSK) receptor is capable of guanylate cyclase activity and enabling cyclic GMP-dependent signaling in plants J Biol Chem 2011 286 22580 22588 10.1074/jbc.M110.168823 21504901 PMC3121402

[B8] Isner JC Nuhse T Maathuis FJ The cyclic nucleotide cGMP is involved in plant hormone signalling and alters phosphorylation of Arabidopsis thaliana root proteins J Exp Bot 2012 63 3199 3205 10.1093/jxb/ers045 22345640 PMC3350932

[B9] Gehring CA Irving HR Natriuretic peptides - a class of heterologous molecules in plants Int J Biochem Cell Biol 2003 35 1318 1322 10.1016/S1357-2725(03)00032-3 12798346

[B10] Maathuis FJ Sanders D Sodium uptake in Arabidopsis roots is regulated by cyclic nucleotides Plant Physiol 2001 127 1617 1625 10.1104/pp.010502 11743106 PMC133566

[B11] Donaldson L Ludidi N Knight MR Gehring C Denby K Salt and osmotic stress cause rapid increases in Arabidopsis thaliana cGMP levels FEBS Lett 2004 569 317 320 10.1016/j.febslet.2004.06.016 15225654

[B12] Pasqualini S Meier S Gehring C Madeo L Fornaciari M Romano B Ederli L Ozone and nitric oxide induce cGMP-dependent and -independent transcription of defence genes in tobacco New Phytol 2009 181 860 870 10.1111/j.1469-8137.2008.02711.x 19140946

[B13] Meier S Madeo L Ederli L Donaldson L Pasqualini S Gehring C Deciphering cGMP signatures and cGMP-dependent pathways in plant defence Plant Sign Behav 2009 4 307 309 10.4161/psb.4.4.8066 PMC2664491 19794847

[B14] Qi Z Verma R Gehring C Yamaguchi Y Zhao YC Ryan CA Berkowitz GA Ca2+ signaling by plant Arabidopsis thaliana Pep peptides depends on AtPepR1, a receptor with guanylyl cyclase activity, and cGMP-activated Ca2+ channels Proc natl Acad Sci USA 2010 107 21193 21198 10.1073/pnas.1000191107 21088220 PMC3000296

[B15] Murashige T Skoog F A revised medium for rapid growth and bioassays with tobacco tissue cultures Plant Physiol 1962 15 473 497 10.1111/j.1399-3054.1962.tb08052.x

[B16] Garavaglia BS Thomas L Gottig N Dunger G Garofalo CG Daurelio LD Ndimba B Orellano EG Gehring C Ottado J A eukaryotic-acquired gene by a biotrophic phytopathogen allows prolonged survival on the host by counteracting the shut-down of plant photosynthesis PLoS One 2010 5 e8950 10.1371/journal.pone.0008950 20126632 PMC2812515

[B17] Eriksson A Bergquist J Edwards K Hagfeldt A Malmstrom D Agmo Hernandez V Optimized protocol for on-target phosphopeptide enrichment prior to matrix-assisted laser desorption-ionization mass spectrometry using mesoporous titanium dioxide Anal Chem 2010 82 4577 4583 10.1021/ac100589j 20443553

[B18] Al-Shahrour F Carbonell J Minguez P Goetz S Conesa A Tarraga J Medina I Alloza E Montaner D Dopazo J Babelomics: advanced functional profiling of transcriptomics, proteomics and genomics experiments Nucl Acids Res 2008 36 W341 346 10.1093/nar/gkn318 18515841 PMC2447758

[B19] Mittler R Vanderauwera S Gollery M Van Breusegem F Reactive oxygen gene network of plants Trends Plant Sci 2004 9 490 498 10.1016/j.tplants.2004.08.009 15465684

[B20] Grant JJ Loake GJ Role of reactive oxygen intermediates and cognate redox signaling in disease resistance Plant Physiol 2000 124 21 29 10.1104/pp.124.1.21 10982418 PMC1539275

[B21] Bolwell GP Bindschedler LV Blee KA Butt VS Davies DR Gardner SL Gerrish C Minibayeva F The apoplastic oxidative burst in response to biotic stress in plants: a three-component system J Exp Bot 2002 53 1367 1376 10.1093/jexbot/53.372.1367 11997382

[B22] Bindschedler LV Dewdney J Blee KA Stone JM Asai T Plotnikov J Denoux C Hayes T Gerrish C Davies DR Peroxidase-dependent apoplastic oxidative burst in Arabidopsis required for pathogen resistance Plant J 2006 47 851 863 10.1111/j.1365-313X.2006.02837.x 16889645 PMC3233234

[B23] Chen YR Deterding LJ Sturgeon BE Tomer KB Mason RP Protein oxidation of cytochrome C by reactive halogen species enhances its peroxidase activity J Biol Chem 2002 277 29781 29791 10.1074/jbc.M200709200 12050149

[B24] Valderrama B Ayala M Vazquez-Duhalt R Suicide inactivation of peroxidases and the challenge of engineering more robust enzymes Chem Biol 2002 9 555 565 10.1016/S1074-5521(02)00149-7 12031662

[B25] Jankovic B Seoighe C Alqurashi M Gehring C Is there evidence of optimisation for carbon efficiency in plant proteomes? Plant Biol 2011 13 831 834 10.1111/j.1438-8677.2011.00494.x 21973021

[B26] Ficarro SB Parikh JR Blank NC Marto JA Niobium(V) oxide (Nb2O5): application to phosphoproteomics Anal Chem 2008 80 4606 46131 10.1021/ac800564h 18491922

[B27] Kwon SJ Kwon SI Bae MS Cho EJ Park OK Role of the methionine sulfoxide reductase MsrB3 in cold acclimation in Arabidopsis Plant Cell Physiol 2007 48 1713 1723 10.1093/pcp/pcm143 17956860

